# Proteomic analysis of the response of *Trichinella spiralis* muscle larvae to exogenous nitric oxide

**DOI:** 10.1371/journal.pone.0198205

**Published:** 2018-06-05

**Authors:** Xiaoli Wang, Liang Li, Xing Wei, Yuanyuan Wang, Hui Zhang, Ao Shi, Tao Liu, Xiaodi Yang, Qiang Fang

**Affiliations:** 1 Department of Microbiology and Parasitology, Anhui Key Laboratory of Infection and Immunity, Bengbu Medical College, Bengbu, China; 2 Department of Parasitology, Medical College of Soochow University, Suzhou, China; 3 Department of Biology and Food Engineering, Bengbu College, Bengbu, China; Institute of Oceanology, Chinese Academy of Sciences, CHINA

## Abstract

*Trichinella spiralis* mainly dwells in the muscle tissue of its host and is the main causative agent of trichinellosis in humans. Nitric oxide (NO), an important intracellular signaling molecule that may restrict pathogen growth in infected hosts, has been known for its anti-pathogenic activity, including resistance to *T*. *spiralis*. Herein, we applied label-free analysis to investigate the effect of sodium nitroprusside (SNP, a NO donor compound) on the proteome of *T*. *spiralis* muscle larvae (ML), followed by Gene ontology (GO) and Kyoto Encyclopedia of Genes and Genomes (KEGG) pathway cluster analyses. Of the 1,476 proteins detected in the ML, 121 proteins showed differential expression, including 50 significantly upregulated and 71 downregulated proteins. The functions of the 108 annotated proteins were primarily related to signal transduction, transcription/translation, material metabolism, protein synthesis/assembly/degradation, and stress/defense/antioxidation. Quantitative real-time polymerase chain reaction (qRT-PCR) assay verified that FRMD5 and CUT-1 gene expression levels were significantly increased, while COX2 gene expression level was significantly decreased. GO annotation and KEGG pathway analyses showed that the majority of differentially expressed proteins were mainly involved in the molecular function of the catalytic activity, biological process of the immune system process, metabolic process, cellular component organization, biological adhesion, and cellular component of the macromolecular complex. Our results demonstrate the first comprehensive protein expression profile of the *ML* in response to NO stress and provide novel references for understanding the potential mechanism underlying the effects of NO on trichinellosis.

## Introduction

*Trichinella* spp. is the smallest, but clinically important, nematode parasite that is widespread and a main causative agent of trichinellosis [1,2]. It is commonly observed in carnivorous mammals and omnivores, including pigs, rodents, and humans [3–5]. Trichinellosis has been reported in about 55 countries worldwide and is very common in developing countries [6,7]. There have been 12 identified genotypes of *Trichinella*. *Trichinella spiralis* is the main etiological agent of most human infections and deaths caused by trichinellosis. From 2004 to 2009, 15 outbreaks of human trichinellosis comprising of 1,387 cases and 4 deaths have been reported in China [8,9]. Trichinellosis is a major food-borne zoonosis transmitted via ingestion of raw or incompletely cooked meat containing infective *Trichinella* larvae, and has health, social, and economic impacts in endemic countries [10]. The life cycle of *T*. *spiralis* includes adult worms, newborn larvae, and muscle larvae (ML) [1,11]. The encapsulation of ML in the skeletal muscles may cause acute phase symptoms such as fever, myalgia, and eosinophilia [12]. During its intimate interaction with the host, *Trichinella* adopts itself to modulate the host immune function for its survival. Studies have shown that excessive reactive oxygen species (ROS) such as peroxide, nitric oxide (NO), and superoxide, are important pathogenic factors of trichinellosis. Although excessive ROS may facilitate the elimination of the parasite, it can also damage tissues and cells [13].

It has been shown that NO serves as a “double duty” molecule in the immune system by acting as an immunotoxin as well as immunomodulator [14]. As a gaseous signaling molecule with significant lipid solubility, NO exerts lethal effects on bacteria and viruses without binding to any specific receptors [15–17] and selectively kills the parasites and infected cells [18]. Lawrence *et al*. reported that NO played an important role in the expulsion of the adult worm during *T*. *spiralis* infections in mice [19]. In addition, free radical-based host defense was shown to be involved in the chemotherapy of trichinellosis [20]. Several previous studies have reported the high expression of inducible nitric oxide synthase (iNOS), especially around cysts, in response to trichinellosis [19–22]. Sodium nitroprusside (SNP), commonly used as an exogenous NO donor compound, may spontaneously release NO in a non-linear manner [23]. The exogenously released NO from SNP was shown to impart significant lethal effects on the adult worm and ML of *T*. *spiralis*; however, the detailed mechanism is still unknown [24].

Proteomics based on mass spectrometry and bioinformatics can be a potent method to elucidate post-translational modifications such as glycosylation or proteolysis. Proteomic techniques have been recently applied to complement genetic studies on different *Trichinella* spp.[25–27]. In the current study, SNP was chosen as an exogenous NO-generating agent [28] to treat ML of *T*. *spiralis*, and the differential expressions of proteins were identified by a label-free analysis coupled with liquid chromatography-tandem mass spectrometry (LC-MS/MS). Furthermore, the transcriptional expressions of genes corresponding to important differentially expressed proteins were verified by a quantitative real-time polymerase chain reaction (qRT-PCR) assay. These results will help to distinguish molecules that were involved in various processes, including redox homeostasis, energy metabolism, protein synthesis, and transcription/translation in response to NO stress. This study provides valuable information for exploring the potential targets and mechanism for controlling trichinellosis.

## Materials and methods

### Parasites and animals

The *Trichinella spiralis* isolate (ISS533) was gifted by Professor Wang from the Department of Parasitology, Medical College, Zhengzhou University, and was maintained by serial passages in BALB/c mice in our laboratory. Specific pathogen-free BALB/c mice (female, 6–8 weeks) were housed in groups of three or four in a climate-controlled room under a 12-h light/dark cycle. All animal experiments were approved by the Institutional Animal Care and Use Committee of Bengbu Medical College and conformed to the current guidelines for the Care and Use of Laboratory Animals published by the U.S. National Institutes of Health (NIH Publication No. 85–23, revised 1996).

### Separation of *T*. *spiralis* ML and treatment with SNP

The infected mice were killed by pentobarbital sodium (35 mg/kg). The muscle larvae of *T*. *spiralis* were recovered from the skeletal muscles of infected mice using the digestion method, as previously described [29]. Muscle larvae separated from all infected mice were pooled and inoculated into a six-well plate in pre-warmed Roswell Park Memorial Institute (RPMI)-1640 medium containing 2 mM L-glutamine and antibiotics (100 U/mL penicillin and 100 μg/mL streptomycin) at 37°C and 5% CO_2_ for 2 h. The larvae with poor activity were removed. SNP (final concentration, 1 mM) was added into the well on day 1 and 2, followed by continuous culture for 3 days to collect live *T*. *spiralis* ML. The control groups were treated without SNP.

### Total protein extraction and analysis by sodium dodecyl sulfate polyacrylamide gel electrophoresis (SDS-PAGE)

The separated *T*. *spiralis* ML were collected and subjected to protein extraction using cell lysis buffer (mainly containing 50 mM Tris [pH = 7.4], 1% Triton X-100, 1% sodium deoxycholate, 0.1% SDS, 1 mM sodium orthovanadate, 1 mM sodium fluoride, 1 mM ethylenediaminetetraacetic acid, and 1 mM protease inhibitor), followed by ultrasonication. The protein concentration was measured using bicinchoninic acid protein assay reagents (Thermo Fisher Scientific Inc.). Equal amounts (~15 μg) of protein samples were separated by SDS-PAGE using 12% polyacrylamide gels and stained with Coomassie Brilliant Blue R-250 (Bio-Rad Laboratories, Inc, USA). The gel was destained using a destaining solution (60% ddH_2_O, 30% ethanol, and 10% acetic acid). Then, images were gathered and analyzed with software (Beijing Sage Creation Science Co., Ltd, Beijing, China).

### Total protein extraction and identification by label-free analysis

The separated *T*. *spiralis* muscle larvae were depleted of the most abundant proteins using ProteoMiner protein enrichment kit (Bio-Rad Laboratories, Inc.) according to the manufacturer’s instructions. Proteins in the resultant elution solutions were precipitated in four volumes of precooled acetone at 20°C. After centrifugation, the protein pellets were air-dried and resuspended in a solution containing 8 M urea and 100 mM triethylammonium bicarbonate (TEAB; pH = 8.0). Protein samples were reduced with 10 mM dithiothreitol (DTT) at 56°C for 30 min, alkylated with 50 mM iodoacetamide for 30 min in the dark, and then diluted four times with 10 mM TEAB. Total protein concentration was measured using the Bradford assay. Equal amounts of proteins for each sample were used for tryptic digestion. Trypsin was added at an enzyme-protein ratio of 1:50 (w/w) and the digestion reaction was performed at 37°C for 12–16 h. After digestion, peptides were desalted using C_18_ columns, and the desalted peptides were dried with a vacuum concentration meter.

The peptide samples were dissolved in 2% acetonitrile/0.1% formic acid and analyzed using a TripleTOF 5600+ mass spectrometer coupled with the Eksigent nano LC System (SCIEX, USA). Peptide samples were loaded onto a C_18_ trap column (5 μm, 100 μm × 20 mm) and eluted at 300 mL/min onto a C_18_ analytical column (3 μm, 75 μm × 150 mm) over a 120-min gradient. The two mobile phases were buffer A (2% acetonitrile / 0.1% formic acid / 98% H_2_O) and buffer B (98% acetonitrile, 0.1% formic acid, 2% H_2_O). For information-dependent acquisition (IDA), survey scans were acquired in 250 ms and 40 product ion scans were collected in 50 ms/scan. MS1 spectra were collected in the range of 350–1,500 m/z, and MS2 spectra were collected in the range of 100–500 m/z. Precursor ions were excluded from the reselection for 15 s.

Raw data were processed using MASCOT (V2.3.02, http://www.matrixscience.com/cgi/search_form.pl?FORMVER=2&SEARCH=MIS). The protein database was referred to the European Biological Information Institute website (http://www.ebi.ac.uk/IPI/). Trypsin/P was specified as cleavage enzyme allowing up to 2 missing cleavage sites. The mass tolerance for precursor ions and daughter ions were set as 5 ppm and 0.5 Da, respectively. Carbamidomethyl on Cys was specified as fixed modification, and complete enzyme peptides were also set as search parameters.

### qRT-PCR assay

Total RNA was used as the template in a reverse transcription reaction using a ReverTra Ace qPCR RT kit (TOYOBO, Japan) according to the manufacturer’s instructions. The reaction mixture, including 10 μL 2× loading buffer, 1.2 μL oligo (dT), 2 μL RNA, 0.2 μL MMLV, and 6.6 μL diethyl pyrocarbonate-treated water, was prepared and subjected to the following program: 65°C for 30 min, followed by 42°C for 30 min and 85°C for 10 min. A total of 100 ng cDNA was subsequently used as the template in an qRT-PCR using SYBR^®^ Premix Ex Taq^TM^ kit (TaKaRa, Cat.RR420A), according to the manufacturer’s instructions. The reaction mixture, including 10 μL 2 × Master Mix, 0.08 μL forward primer, 0.08 μL reverse primer, 2 μL cDNA, 0.4 μL Taq DNA polymerase, and 7.44 μL ddH_2_O, was prepared and subjected to qRT-PCR analysis as per the following program: one cycle of 95°C for 3 min, followed by 40 cycles of 95°C for 12 s, 62°C for 30 s, and 72°C for 30 s. The primers used are shown in S2 Table. The results were analyzed using SDS 1.4 software (Applied Biosystems) based on the 2^−ΔΔCt^ method, and histogram analyses using the Origin 9.5 software (http://www.originlab.com/).

### Gene ontology (GO) clustering

The GO database (http://geneontology.org/) includes three functional categories: biological process, cellular component, and molecular function. Genes may be further organized by directed acyclic graph according to their scopes. In GO clustering, genes are considered significantly enriched based on the ratio of the observed GO term for all genes to the GO term for a single gene set. First, each gene that was assigned a particular GO term was broadly noted in the upper father node, and the p value of each enriched GO term was determined using a hypergeometric distribution. The p value was adjusted using a false discovery rate (FDR), with p = 0.05 selected as the threshold value. The redundant GO terms were subsequently removed and the hierarchy chart’s terminal nodes were selected as the final significantly enriched GO terms.

### Kyoto Encyclopedia of Genes and Genomes (KEGG) pathway clustering

The Kyoto Encyclopedia of Genes and Genomes database (http://www.genome.jp/kegg/pathway.html) was used to systematically analyze gene function and genomic information from biological pathways and to further categorize biological pathways according to metabolism, enzyme, biochemical reaction, gene regulation, and protein–protein interactions. Here, KEGG signaling pathway analysis was applied, followed by a hypergeometric distribution analysis and a FDR method to yield an adjusted p value (p = 0.05 as a threshold value).

### Statistical analysis

All data are expressed as the mean ± standard deviation (SD). Statistical analysis was performed with the *t*-test using the SPSS software (version 21.0, http://spss.en.softonic.com/; Chicago, IL, USA). Student′s *t*-test was performed in a group of two samples. A value of p < 0.05 and p < 0.01 was considered to be significant and highly significant, respectively.

## Results

### Differential protein expression analysis by SDS-PAGE

**Fig 1:**
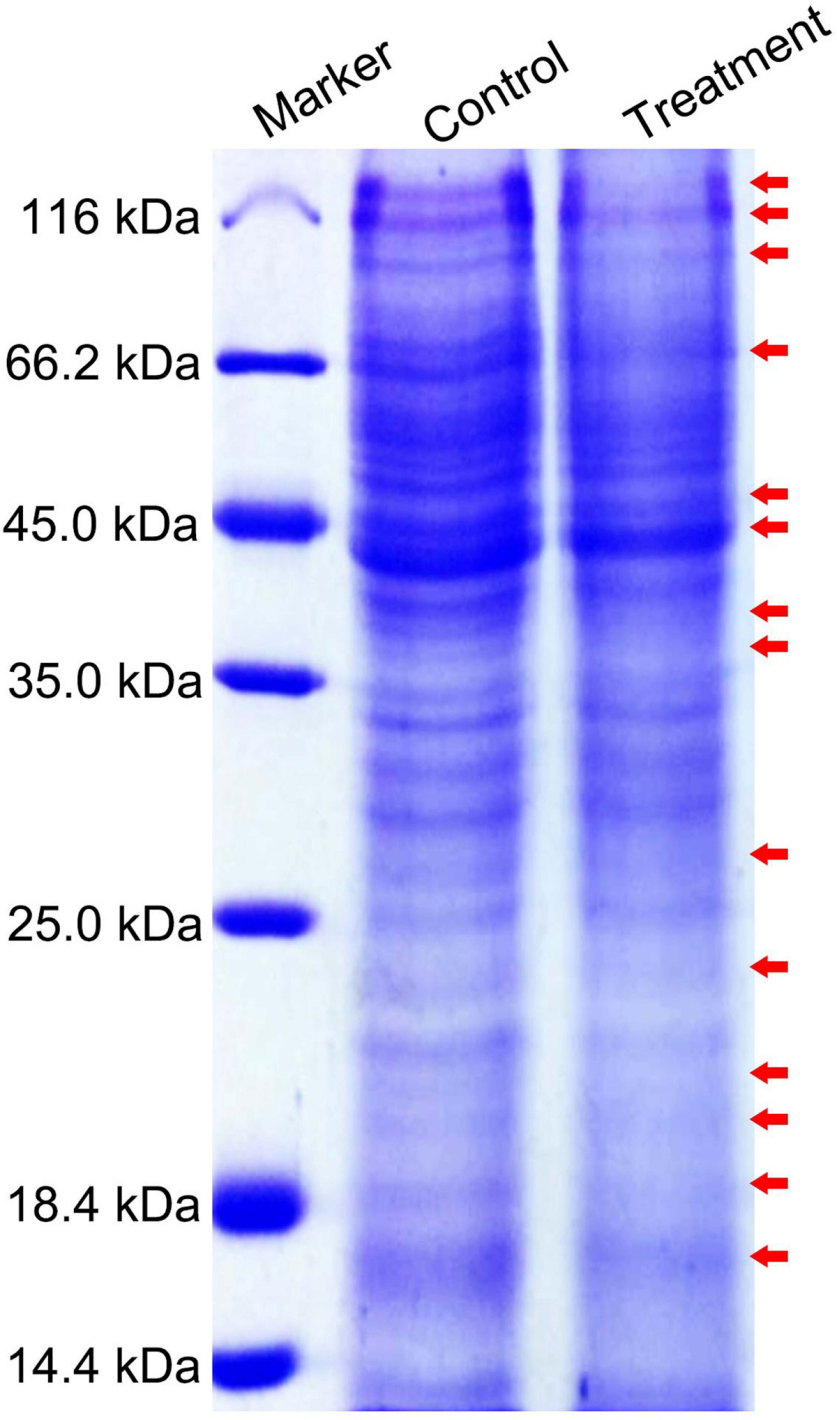
SDS-PAGE analysis of the proteins from *T*. *spiralis* muscle larvae. The images indicated that several different proteins (arrow) were identified via 12% SDS-PAGE.

Several different protein bands were identified from *T*. *spiralis* ML after SNP treatment as compared with the control group (Fig 1). Thus, different protein expression profiles were observed by SDS-PAGE analysis.

### Differential protein expression profiles by label-free analysis after SNP treatment of *T*. *spiralis* ML

As shown in Table 1 and S1 Fig, the label-free analysis revealed a total of 92,142 spectra (including 48,740 and 43,402 spectra in the control and treatment groups, respectively), 10,320 peptides (including 8,579 and 8,615 peptides in the control and treatment groups, respectively), and 1,476 proteins (including 1,310 and 1,293 proteins in the control and treatment groups, respectively).

**Table 1:** Summary of the identified proteins from *T*. *spiralis* muscle larvae after sodium nitroprusside treatment.

Type	Control	Treatment	All Combined
**Spectrum**	48,740	43,402	92,142
**Peptides**	8,579	8,615	10,320
**Proteins**	1,310	1,293	1,476

The distribution of the quality tolerance was in a range of ±0.1 Da and indicated higher accuracy. The distribution of peptide length was focused in a range of 8–22, in accordance with the molecular scope (400–1,200) of mass spectrometer identification (S2 Fig). In addition, the unique peptide distribution of the identified proteins were shown in S2 Fig. A correlation analysis showed that the r value of Pearson's correlation was 0.939, and a higher correlation was observed in the control and treatment groups (S3 Fig).

A total of 121 differentially expressed proteins, including 71 and 50 significantly downregulated and upregulated proteins, respectively, were identified after a label-free analysis. Of these 121 proteins, 108 proteins were annotated and mainly involved in signal transduction, energy metabolism, protein synthesis/assembly/degradation, and stress/defense/antioxidation (S1 Table). The peptide mass fingerprinting of the five significantly upregulated and downregulated proteins, respectively, were shown in S4 Fig.

### qRT-PCR analysis of differentially expressed proteins

**Fig 2:**
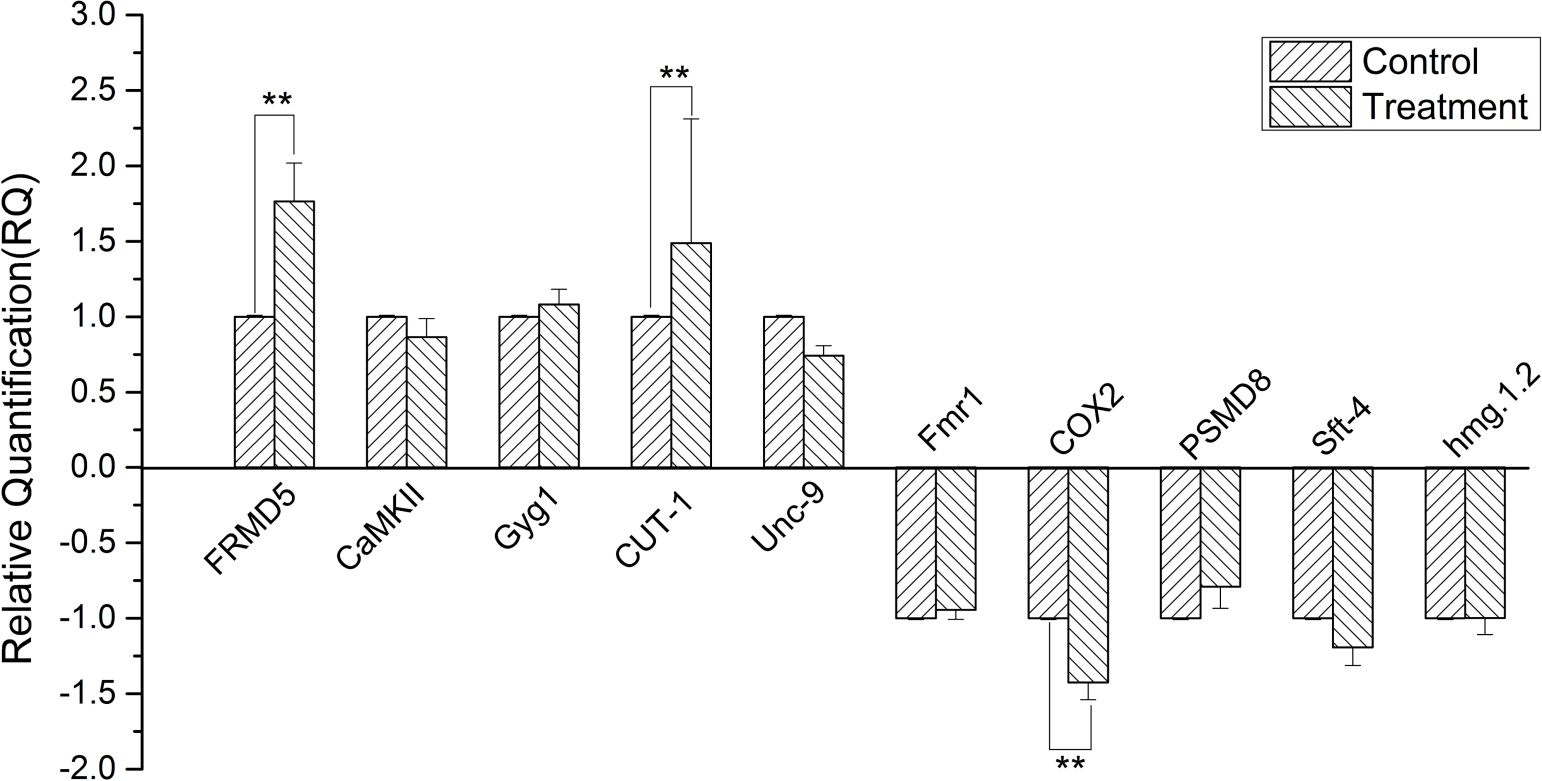
qRT-PCR assay of *T*. *spiralis* ML after SNP treatment. The images indicated that FRMD5 and CUT-1 gene expression levels were significantly increased (**: *p* < 0.01, compared to control), and COX2 gene expression levels were significantly decreased (**: *p* < 0.01, compared to control). The gene abbreviations and their corresponding protein IDs (Uniprot accession numbers) were presented below. FRMD5 (A0A0V1B0F8), FERM domain-containing protein 5; Gyg1 (A0A0V1BIX6), glycogenin-1; CaMKII (A0A0V1B330), calcium/calmodulin-dependent protein kinase type II alpha chain; CUT-1 (A0A0V1BRH2), cuticlin-1; Unc-9 (A0A0V1BCD9), innexin; Fmr1(A0A0V1BBL6), fragile X mental retardation protein 1-like protein B; PSMD8 (A0A0V1B4G3), 26S proteasome non-ATPase regulatory subunit 8; hmg.1.2(A0A0V1BWL9), high mobility group protein 1.2; sft-4 (E5S1B0), surfeit locus protein 4; and COX2(Q9B8A2), cytochrome c oxidase subunit 2.

To experimentally verify the accuracy of differentially expressed proteins by label-free proteomic analysis, we chose 10 genes for the qRT-PCR analysis and quantified their transcript levels. The results suggested that the identified proteins were regulated at the transcriptional level. As shown in Fig 2, five upregulated proteins, including FRMD5, CaMKII, Gyg1, CUT-1, and Unc-9, and five downregulated proteins, including Fmr1, COX2, PSMD8, Sft-4, and hmg.1.2, were identified by qRT-PCR using primers shown in S2 Table. Three genes (FRMD5, CUT-1, and COX2) showed expression patterns similar to their protein levels, while other genes displayed no statistical differences. FRMD5 and CUT-1 expression levels were significantly increased after SNP treatment of *T*. *spiralis* ML (**, *p* < 0.01). However, COX2 expression level was significantly lower in the treatment group than in the control group (**, *p* < 0.01).

### GO cluster analysis of the differentially expressed proteins after SNP treatment

**Fig 3:**
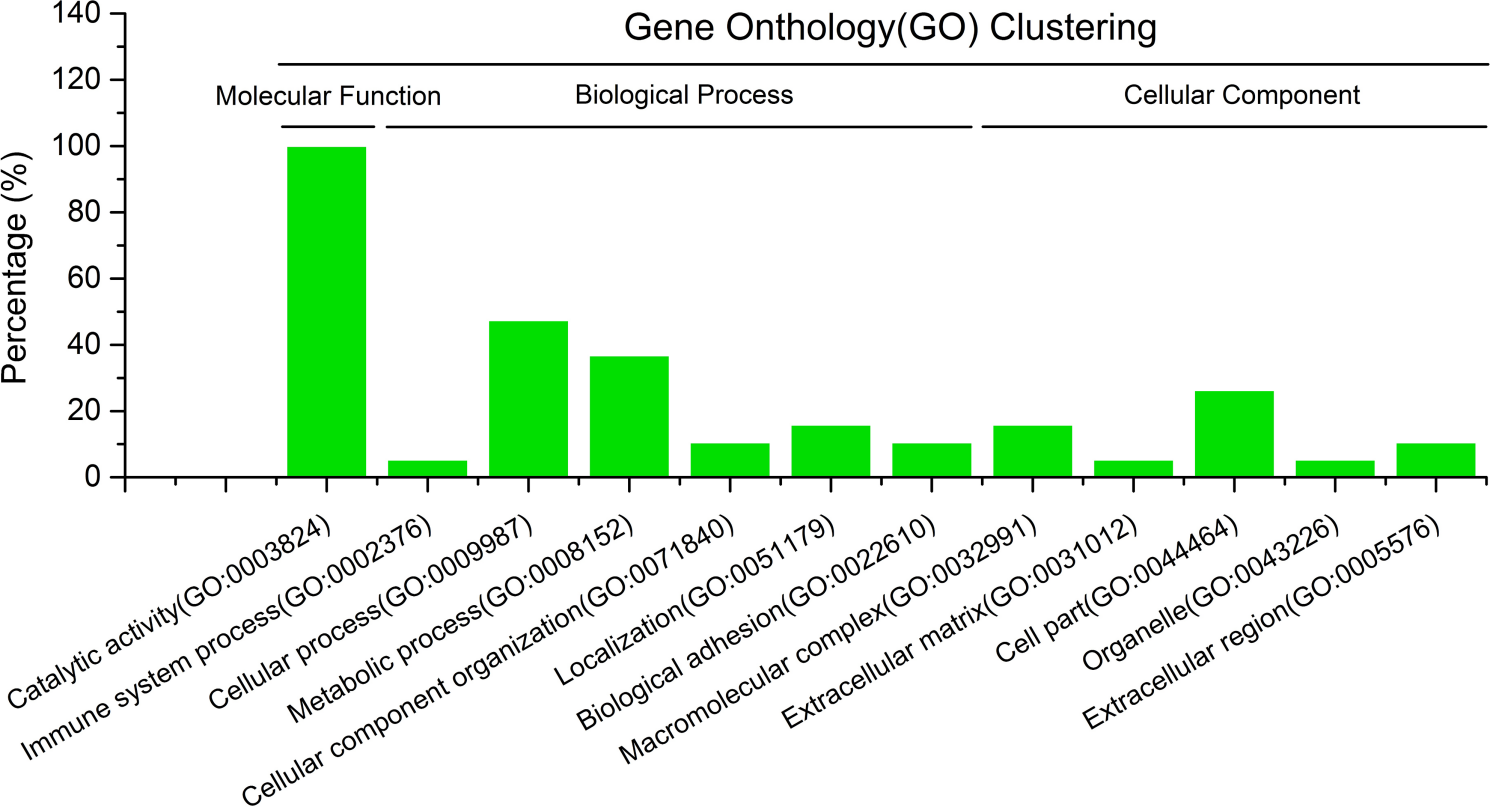
Gene ontology analysis of differentially expressed proteins from *T*. *spiralis* muscle larvae. The images indicated that the differentially expressed proteins involved in the molecular function of catalytic activities; biological processes such as immune system processes, cellular processes, metabolic processes, cellular component organization, localization, and biological adhesion; and the cellular component of the macromolecular complex, extracellular matrix, cell part, organelle, and extracellular region. These proteins exhibited roles in several significant biological processes.

As shown in Fig 3, the differentially expressed proteins in *T*. *spiralis* ML after SNP treatment were mainly associated with the molecular functions of catalytic activities; biological processes such as immune system processes, cellular processes, metabolic processes, cellular component organization, localization, and biological adhesion; and cellular component of the macromolecular complex, extracellular matrix, cell part, organelle, and extracellular region. These proteins exhibited roles in several significant biological processes.

### Analysis of differentially expressed proteins from *T*. *spiralis* ML by KEGG pathway

**Fig 4:**
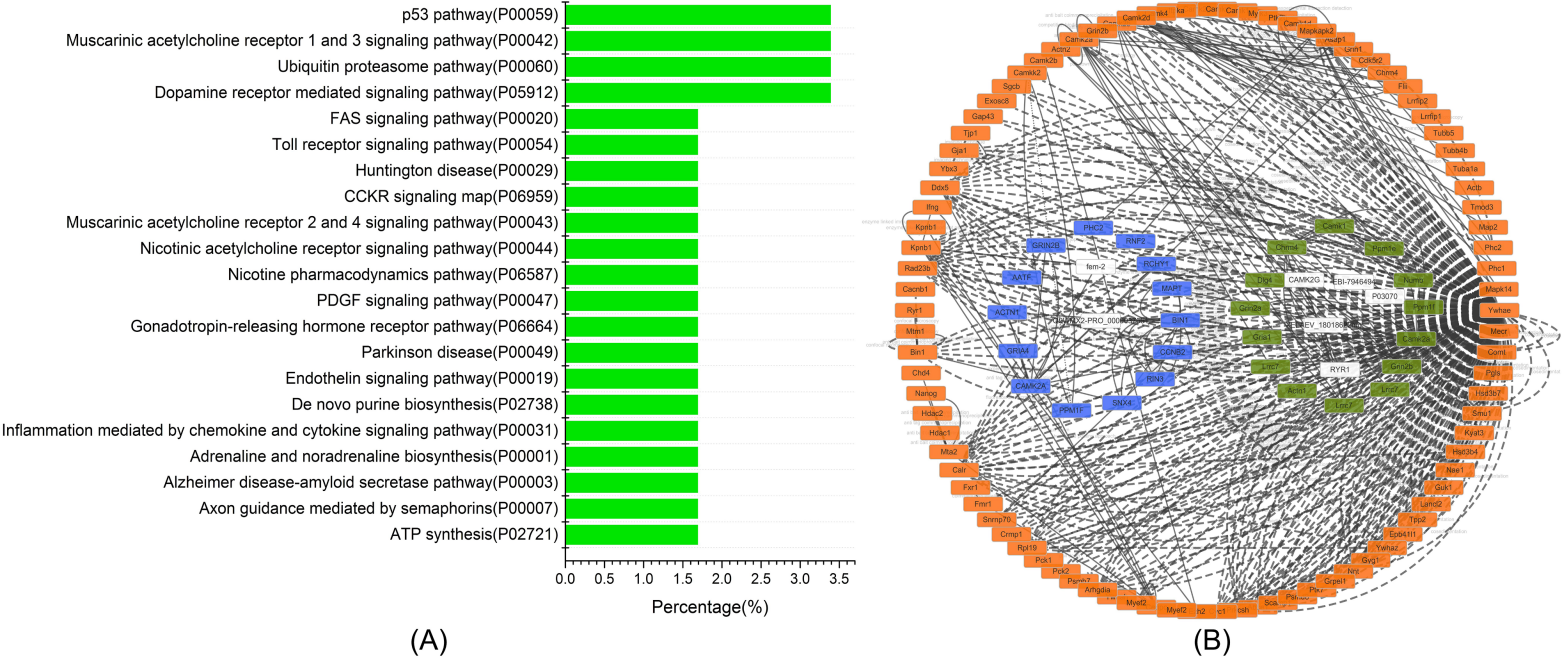
KEGG pathway analysis of differential expression proteins associated genes. (A) Histogram analysis of the KEGG pathway of differentially expressed proteins for *T*. *spiralis* muscle larvae after sodium nitroprusside treatment. (B) Cytoscape software analysis of the pathway of differentially expressed proteins in *T*. *spiralis* ML after SNP treatment. The images indicated that several important differentially expressed genes were involved in various significant signal pathways.

The KEGG pathway analysis revealed that the most significant pathways in response to NO stress included p53, ubiquitin proteasome, muscarinic acetylcholine receptor 1 and 3, and dopamine receptor-mediated signaling pathways. In addition, axon guidance mediated by semaphorins, Alzheimer disease-amyloid secretase pathway, adrenaline and noradrenaline biosynthesis, inflammation mediated by chemokine and cytokine signaling pathways, *de novo* purine biosynthesis, endothelin signaling pathway, Parkinson’s disease, gonadotropin-releasing hormone receptor pathway, PDGF signaling pathway, nicotine pharmacodynamics pathway, nicotinic acetylcholine receptor signaling pathway, muscarinic acetylcholine receptor 2 and 4 signaling pathway, CCKR signaling map, Huntington’s disease, ATP synthesis, toll receptor signaling pathway, and FAS signaling pathway were involved (Fig 4A). PSTK-regulated mRNAs were further analyzed by Cytoscape software (Fig 4B); the differentially expressed proteins in *T*. *spiralis* ML in response to NO stress were involved in several complex networks.

## Discussion

The parasite *T*. *spiralis*, one of the most widespread and clinically important parasites in the world, has an unusual life cycle. The adult worms mature in the small intestine of a definitive host, while the new larvae drill into the intestinal wall, then enters the blood (to feed on it) or lymphatic system and striated muscle to enclose into a capsule [30]. The migration and encystment of the larvae may cause variable clinical manifestations. In some cases, accidental migration to specific organs may cause myocarditis and encephalitis, leading to death [31]. Successful parasitism mainly depends on the host and the parasite. In the context, immunoregulatory cytokine, such as NO, could be closely related to the survival of the parasite in *Trichinella*-infected host [32]. As a crucial mediator, NO can restrict pathogen growth in infected animals, which has been shown direct toxicity to the worm in *vitro* [33,34]. In addition, NO plays a role as important immune effectors in the pathogenesis of trichinellosis induced by either Th1 or Th2 responses [19,35]. However, based on NO expulsion of *T*. *spiralis*, there have been no curated or predicted related pathogen genes from the parasite that have been identified, and the exact underlying mechanism remains elusive. Proteomic analysis is a powerful tool to screen samples derived from pathogens for the identification of proteins possibly involved in pathogenesis [1,27,36]. The expansion of sequence databases for *T*. *spiralis* has provided new opportunities for proteomic analysis of trichinellosis to gain a better understanding of parasite biology and host-parasite interactions [37,38]. Therefore, SNP was chosen as the exogenous NO-generating agent [39] to treat *T*. *spiralis* ML *in vitro* and for the evaluation of the mechanism underlying NO-mediated resistance to ML.

In this study, the proteomic expression profile of *T*. *spiralis* ML in response to NO stress was determined by label-free analyses coupled with LC-MS/MS. We identified 1,476 proteins, of which 121 were differentially expressed. The differentially expressed proteins were mainly involved in the molecular function of catalytic activities; biological processes such as immune system process, cellular process, and metabolic process; and cellular components of the macromolecular complex, cell part, and organelle. The functions of the 108 annotated proteins were primarily related to protein synthesis/assembly/degradation, signal transduction, transcription/translation, material metabolism, and stress/defense/antioxidation. We used a qRT-PCR assay to verify that FRMD5, CUT-1, and COX2 gene expression levels were statistically significant between the two groups, consistent with the results of the high-throughput sequencing.

The protein TspA0A0V1B0F8 was FERM domain-containing protein 5 and showed a statistically significant change after SNP treatment. FERM superfamily members display a conserved FERM (protein four-point-one, Ezrin, Radixin, Moesin) domain [40]. This superfamily comprises several subgroups in humans, including FERM proteins, ERM, MyTh-myosin, Janus kinases, and FERM-FA. FERM domain-related sequences have also been identified in plants, fungi, unicellular eukaryotes, and *Drosophila* [41]. FRMD5, similar to FRMD3, has been reported as a FERM and FA domain-containing novel tumor suppressive molecule, which may maintain cell-cell contact and regulate tumor progression. In addition, FRMD5 is known as a potential target of p53^(R273H)^ [42]. Guo *et al*. reported that the FRMD5 gene was associated with lipid homeostasis [43]. It has been demonstrated that iNOS overexpression or NO donors increased cell death receptor expression and upregulated the p53 pathway [44]. In our study, the p53 pathway and metabolic processes were most affected and were accompanied with an increase in FRMD5 gene expression after treatment of *T*. *spiralis* ML with NO. This result may provide new insight into the relationship between NO, the p53 pathway, and FERM proteins. Furthermore, it may be possible for the administration of a NO donor as a potential chemotherapeutic candidate against trichinellosis.

The cuticle around the worm is a tough, elastic, extracellular layer, which is secreted by the underlying epithelia. It acts as an exoskeleton, participates in nutrient absorption, determines the shape of the worm, and helps in movement. In addition, the cuticle regulates the interactions of the worm with the environment, confers the protection, and allows the growth [45]. Cuticlin is an insoluble residue remaining from the cuticle and is mainly composed of various heavily cross-linked non-collagenous compounds. CUT-1 protein was the first identified cuticlin and contained a zona pellucida (ZP) domain [46]. CUT-1-like proteins have been reported as important components for eliciting the host immune response to invading nematodes. CUT-1-like proteins have been identified in nematodes such as *Caenorhabditis elegans*, *Ascaris lumbricoides*, and *Heterorhabditis* [47]. There has been enormous evidence demonstrating that parasites manipulate the host immune response to prolong their survival [33]. In the current study, we observed a consistent increase in the expression and transcription of CUT-1-like proteins (TspA0A0V1BRH2). It was therefore possible that the increasing expression of CUT-1-like proteins were involved in the protective mechanism, meanwhile confirming an essential role for the cuticle, which helped ML escape from NO-mediated oxidative stress, and highlighting its potential targets.

Cytochrome c oxidase (COX) is a major enzyme of the mitochondrial respiratory chain and a major oxygen consumer in the cell. Several regulatory pathways converge to facilitate efficient COX assembly, thereby prevent oxidative stress. In eukaryotes, the respiratory enzymatic machinery is central to energy conversion and is accompanied with ROS as an inevitable by-product. COX has a nuclear-encoded subunit, which allows the organism to adapt to the environmental oxygen levels (Cox5 in yeast and COX4 in mammals). Cox5/COX4 has two core subunits, Cox1 and Cox2, which affect COX redox centers and holoenzyme stability [48]. The mitochondrial COX1 and COX2 genes are widely applied to identify parasite species and to distinguish evolutionary relationships between parasites. This gene has been evaluated as a potential genetic marker in the phylum of parasites such as *Aulonocephalus pennula*, *Contracaecum osculatum*, and *Cysticercus tenuicollis* [49,50]. It has been documented that NO is toxic for *T*. *spiralis* newborn and growing larvae [33,34]. In our study, the protein and mRNA levels of COX2 were lower in the SNP-treated group than in the control group. It was possible that there was a lack of ROS elimination in the environment, amount of NO inhibiting COX2 gene, and decreasing COX2 protein expression, and even damaged to muscle larvae or lethality to the worms. These findings suggested that COX2 played a crucial role in the ML redox-regulation, and provided a reference for further exploring NO mechanism in the host-parasite relationship.

GO analysis of protein functions showed that the catalytic activity was the key molecular function associated with NO treatment of ML. The comparison between the two groups showed that some proteins (e.g., serine protease, DNase II superfamily, and cystatin) exhibited high differential expression. These proteins may be related to *T*. *spiralis* invasion in cells and growth development of the parasite, and played important roles in a variety of physiological activities. Serine proteases are involved in many processes such as digestion, coagulation, and fibrinolysis [51]. In parasites, serine proteases have been shown to be involved in functions beneficial for host tissue invasion, such as moulting, changes in cell surface antigens, and degradation of the intercellular or cytoplasmic proteins [52]. The TspSP-1.2 recombinant protein may induce immunological protection in mice and was considered to be a preventive vaccine candidate for trichinellosis [53]. Enzymes of the DNaseII family and lysosomal proteins may induce the formation of supercoiled plasmids from a single or double DNA chain and mediate cell apoptosis [54]. Reports have shown the abundant secretion of cystatin in nematodes and its role in the invasion of the host tissue [55]. In the current study, the changes in serine proteases (TspE5SE12), DNaseII family (TspQ27073), and cystatin (TspA1BQX7) expression were indicative of the suppression of *T*. *spiralis* ML invasion after NO treatment.

We found that the proteins involved in material metabolism, signal transduction, stress/defense/antioxidation, and transcription/translation were obviously affected. In addition, GO and KEGG pathway analyses showed that the differentially expressed proteins were involved in other signaling pathways such as the p53 pathway, ATP synthesis, ubiquitin proteasome pathway, and *de novo* purine biosynthesis. The upregulation or increase in protein expression may protect the worm under NO stress and help it adapt to the oxidative stress. On the other hand, the downregulation of protein expression was an indication of the damage mediated by oxidative reactions. Understanding the mechanism by which NO interferes with *T*. *spiralis* would be extremely useful for future research. In summary, our work established a comprehensive proteomic analysis of *T*. *spiralis* ML, and analyzed the molecular processes involved in the complicated regulatory networks responding to NO stress. However, our study only detected the quantitative protein profiles and mRNA expression levels of differential genes. As these genes showed no association with antibodies, future work should be directed to prepare these antibodies to confirm the expression of different proteins. In addition, although numerous proteins had been identified in this study by mean of mass spectrometry, some different protein points were not matched in the databases. This observation may be associated with the low abundance of these proteins or incomplete protein peptide fingerprint database; there may be some unknown proteins. The functions and interactions of the different proteins identified in this study need further evaluation.

## Conclusions

Based on label-free proteomics techniques, 1,476 proteins were detected in ML in response to NO stress, and a total of 121 differentially expressed proteins were identified, including 50 upregulated and 71 downregulated proteins. The 108 annotated proteins were primarily related to signal transduction, transcription/translation, material metabolism, protein synthesis/assembly/degradation, and stress/defense. Three genes, including FRMD5, CUT-1, and COX2, showed expression patterns similar to their corresponding protein levels. These results may provide valuable references for understanding the molecular processes underlying the effect of NO stress and elucidate the potential mechanisms involved in NO-mediated resistance to ML.

## Supporting information

S1 Table The differential expression profile of *T*. *spiralis* ML after sodium nitroprusside treatment. (DOCX)

S2 Table The primers used in this study. (DOCX)

S1 Fig Label-free analysis of proteins profile from *T*. *spiralis* ML. (A) Proteins expressed in the control group; (B) Proteins expressed in the sodium nitroprusside treatment group. The images indicated that several differential peaks were identified in *T*. *spiralis* muscle larvae in response to NO stress.
(TIF)

S2 Fig Analysis of the expressed proteins from *T*. *spiralis* muscle larvae. (A) Distribution of precursor tolerance. (B) Distribution of peptides length. (C) Unique peptide distribution of the identified proteins. The images indicated that the quality tolerance was in the range of ±0.1 Da, and peptides length was focused on the range of 8–22.
(TIF)

S3 Fig Correlation analysis of differentially expressed proteins. The images indicated that the r of Pearson's correlation was 0.939, and had a higher correlation between the control and treatment groups.
(TIF)

S4 Fig Liquid chromatography-tandem mass spectrometry was used to identify differentially expressed proteins from *T*. *spiralis* ML after sodium nitroprusside treatment. Secondary mass spectrogram of five expressed proteins in the control (A–E) and the treatment groups (F–J), respectively.
(TIF)
