# Supplementary material for: Proteomic analysis of the response of Trichinella spiralis muscle larvae to exogenous nitric oxide
Source: PLoS One. 2018 Jun 5;13(6):e0198205. doi: 10.1371/journal.pone.0198205 (PMC5988324; doi:10.1371/journal.pone.0198205)
Supplement: S1 Table — (DOCX) [file pone.0198205.s001.docx]

Table S1. The differential expression profile of *T. spiralis* ML after SNP treatment

| **Entry name** | **Protein names** | **Gene names** | **Treatment_Abund** | **Control_Abund** | **FoldChange**  **(Treatment: Control)** |
| --- | --- | --- | --- | --- | --- |
| A0A0V1B0F8_TRISP | FERM domain-containing protein 5 | FRMD5 | 5798575 | 999506 | 5.8014 |
| A0A0V1BIX6_TRISP | Glycogenin-1 | Gyg1 | 563324 | 126340 | 4.4588 |
| A0A0V1B330_TRISP | Calcium/calmodulin-dependent protein kinase type II alpha chain | CaMKII | 4606949 | 1097166 | 4.1990 |
| A0A0V1AXH3_TRISP | Dihydropyrimidinase-related protein 1 | Crmp1 | 130612942 | 31686768 | 4.1220 |
| E5SLY1_TRISP | Putative ankyrin repeat and FYVE domain-containing protein 1 | Tsp_08206 | 2585980 | 639604 | 4.0431 |
| A0A0V1B0B1_TRISP | V-type proton ATPase subunit H | VhaSFD | 2524696 | 630886 | 4.0018 |
| E5S9N3_TRISP | Insulin-degrading enzyme | Tsp_00424 | 7758189 | 2121507 | 3.6569 |
| A0A0V1BUP6_TRISP | Somatomedin-B and thrombospondin type-1 domain-containing protein | SBSPON | 20135228 | 5547600 | 3.6295 |
| E5SKA4_TRISP | Signal peptidase subunit superfamily | Tsp_09262 | 7086000 | 1970484 | 3.5961 |
| A0A0V1BRH2_TRISP | Cuticlin-1 | CUT-1 | 3712522 | 1045446 | 3.5511 |
| E5STE0_TRISP | NADH dehydrogenase [ubiquinone] 1 beta subcomplex subunit 7 | T01_9694 | 1556669 | 439396 | 3.5427 |
| A0A0V1BZB9_TRISP | Putative ATP-dependent RNA helicase DDX5 | DDX5 | 1973258 | 565709 | 3.4881 |
| A0A0V1B5T1_TRISP | Vesicle-fusing ATPase 1 | comt | 29058677 | 8332242 | 3.4875 |
| A0A0V1BVU3_TRISP | Nedd8-activating enzyme E1 regulatory subunit | Nae1 | 4412461 | 1317530 | 3.3490 |
| A0A0V1BXD0_TRISP | ATP-binding cassette sub-family E member 1 | Abce1 | 28734937 | 9013559 | 3.1880 |
| A0A0V1B424_TRISP | Myelin expression factor 2 | Myef2 | 35113390 | 11224870 | 3.1282 |
| E5S7F3_TRISP | Uncharacterized protein | T01_7588 | 28385392 | 9111398 | 3.1154 |
| A0A0V1B975_TRISP | Band 4.1-like protein 1 | Epb41l1 | 7133897 | 2350463 | 3.0351 |
| A0A0V1BCD9_TRISP | Innexin | unc-9 | 5484918 | 1809599 | 3.0310 |
| E5ST22_TRISP | Sorting nexin-2 | Tsp_05643 | 3271220 | 1090676 | 2.9993 |
| A0A0V1C066_TRISP | Ubiquitin fusion degradation protein 1-like protein | T01_8520 | 9246489 | 3239811 | 2.8540 |
| E5SMQ3_TRISP | Uncharacterized protein | Tsp_08613 | 6702132 | 2456424 | 2.7284 |
| A0A0V1BUC5_TRISP | Histone deacetylase complex subunit SAP18 | Bin1 | 10584136 | 4063333 | 2.6048 |
| A0A0V1BKJ7_TRISP | Tripeptidyl-peptidase 2 | Tpp2 | 6045393 | 2374146 | 2.5463 |
| A0A0V1BEK6_TRISP | Cytochrome c1, heme protein, mitochondrial | CYC1 | 22973751 | 9101551 | 2.5242 |
| A0A0V1B3D0_TRISP | Cuticle collagen lon-3 | lon-3 | 2394554 | 966498 | 2.4776 |
| E5S659_TRISP | Proteasome subunit beta type-6 | Tsp_06816 | 28305233 | 11477246 | 2.4662 |
| A0A0V1B2K0_TRISP | F-actin-capping protein subunit alpha | cpa | 4752697 | 1930216 | 2.4623 |
| A0A0V1B161_TRISP | Guanylate kinase | Guk1 | 2749128 | 1125123 | 2.4434 |
| E5SGY8_TRISP | Putative small nuclear ribonucleo protein G | snr-7 | 28586285 | 11834388 | 2.4155 |
| A0A0V1C0W7_TRISP | Phosphatidylinositol phosphatase | Ptprq | 87569994 | 36511920 | 2.3984 |
| A0A0V1C195_TRISP | Phosphatidylinositol phosphatase PTPRQ | Ptprq | 4772461 | 2064570 | 2.3116 |
| E5SSW6_TRISP | Putative thioredoxin | Tsp_05590 | 1155591 | 503551 | 2.2949 |
| A0A0V1C2B4_TRISP | Uncharacterized protein | T01_8097 | 5939655 | 2597528 | 2.2867 |
| A0A0V1AVV5_TRISP | Peptidase inhibitor R3HDML | R3HDML | 72468199 | 31765163 | 2.2814 |
| A0A0V1BP90_TRISP | 26S proteasome non-ATPase regulatory subunit 4 | Pros54 | 12240984 | 5386819 | 2.2724 |
| E5S6G0_TRISP | 26S proteasome non-ATPase regulatory subunit 10 | Tsp_06911 | 3238998 | 1440750 | 2.2481 |
| E5S2F4_TRISP | Putative membrane protein | Tsp_03019 | 4710773 | 2099188 | 2.2441 |
| A0A0V1BZZ5_TRISP | H/ACA ribonucleoprotein complex subunit 4 | Nop60B | 6567673 | 2926832 | 2.2440 |
| E5SN08_TRISP | GTP-binding protein Rab-3 | rab-3 | 3587483 | 1599847 | 2.2424 |
| A0A0V1BEH8_TRISP | LIM domain-containing protein unc-97 | unc-97 | 6396833 | 2860998 | 2.2359 |
| A0A0V1BHU1_TRISP | Translation initiation factor eIF-2B subunit delta | Eif2b4 | 2540158 | 1153795 | 2.2016 |
| A0A0V1B8Q5_TRISP | Proteasome subunit beta type-7 | Psmb7 | 8097820 | 3719121 | 2.1773 |
| A0A0V1ATB7_TRISP | Basement membrane proteoglycan | unc-52 | 32514169 | 14999721 | 2.1677 |
| E5SAS1_TRISP | UMP-CMP kinase | Tsp_00848 | 10674599 | 4952331 | 2.1555 |
| E5S5M3_TRISP | Isocitrate dehydrogenase [NADP] | Tsp_06181 | 25419610 | 11956171 | 2.1261 |
| A0A0V1BPX9_TRISP | Kynurenine--oxoglutarate transaminase 3 | CCBL2 | 10775848 | 5105330 | 2.1107 |
| A0A0V1AUS6_TRISP | Inactive tyrosine-protein kinase 7 | PTK7 | 3955894 | 1880875 | 2.1032 |
| A0A0V1B800_TRISP | Importin subunit beta-1 | KPNB1 | 17712289 | 8453125 | 2.0954 |
| E5SNC7_TRISP | Transmembrane emp24 domain-containing protein 1 | tmed1 | 1902006 | 914541 | 2.0797 |
| A0A0V1BA72_TRISP | Calreticulin | CALR | 4268625 | 8555276 | 0.4989 |
| A0A0V1AWD5_TRISP | Chloride intracellular channel exc-4 | exc-4 | 6354621 | 12744685 | 0.4986 |
| A0A0V1BUL8_TRISP | Eukaryotic translation initiation factor 3 subunit C | eif3c | 9011497 | 18089332 | 0.4982 |
| E5S8J5_TRISP | Cysteine and glycine-rich protein 1 | Tsp_00064 | 14251969 | 28665090 | 0.4972 |
| A0A0V1BJ45_TRISP | Uncharacterized protein | T01_10510 | 27751867 | 55877421 | 0.4967 |
| E5SR45_TRISP | Adenylate kinase isoenzyme 6 homolog | Tsp_09539 | 1743354 | 3512265 | 0.4964 |
| E5S2D2_TRISP | Dipeptidyl-peptidase 1 | Tsp_02997 | 1457269 | 2942530 | 0.4952 |
| A0A0V1BX59_TRISP | Integrin alpha pat-2 | pat-2 | 21733624 | 44151575 | 0.4923 |
| E5SKD7_TRISP | V-type proton ATPase subunit C | Tsp_09296 | 1910921 | 3889442 | 0.4913 |
| E5S3P4_TRISP | Uncharacterized protein | Tsp_03447 | 1139268 | 2325616 | 0.4899 |
| A0A0V1BU25_TRISP | ADP-ribosylation factor-like protein 1 | Arf72A | 1758821 | 3601388 | 0.4884 |
| A0A0V1BWX2_TRISP | Venom allergen 5 | T01_3621 | 116807390 | 239284946 | 0.4882 |
| E5SSE2_TRISP | Putative ADP-dependent glucokinase | Tsp_05422 | 1694165 | 3485003 | 0.4861 |
| A0A0V1BUA3_TRISP | Uncharacterized protein | T01_4307 | 5437014 | 11189387 | 0.4859 |
| E5RYV9_TRISP | Putative Transmembrane protease serine 9 | Tmprss9 | 14860751 | 30865220 | 0.4815 |
| E5SH18_TRISP | Alpha-soluble NSF attachment protein | Tsp_03743 | 5620528 | 11694368 | 0.4806 |
| E5SE12_TRISP | Putative trypsin | Tsp_01973 | 44820193 | 93794654 | 0.4779 |
| A0A0V1C0Z6_TRISP | Uncharacterized protein | T01_7410 | 74975667 | 157673474 | 0.4755 |
| A0A0V1B325_TRISP | Uncharacterized protein | T01_11957 | 171211383 | 365246858 | 0.4688 |
| E5S9X7_TRISP | Uncharacterized protein | Tsp_00551 | 54521584 | 116931736 | 0.4663 |
| A0A0V1BIW5_TRISP | Elongation factor 1-alpha | T01_9324 | 1973182 | 4261514 | 0.4630 |
| A0A0V1B563_TRISP | Protein pad-1 | pad-1 | 680083 | 1473945 | 0.4614 |
| A0A0V1BA13_TRISP | Eukaryotic translation initiation factor 3 subunit L (eIF3l) | eif-3.L | 3548150 | 7727536 | 0.4592 |
| A0A0V1ARH0_TRISP | Uncharacterized protein | T01_9266 | 52022490 | 113374797 | 0.4589 |
| A0A0V1BWC3_TRISP | Calcium-binding protein 39-like | CAB39L | 611264 | 1333656 | 0.4583 |
| A0A0V1AWZ3_TRISP | DnaJ-like protein subfamily C member 25 | dnajc25 | 4084627 | 8949183 | 0.4564 |
| A0A0V1C2C9_TRISP | Thiosulfate sulfurtransferase/rhodanese-like domain-containing protein 3 | Tstd3 | 8987071 | 19735999 | 0.4554 |
| A0A0V1BSQ2_TRISP | Glucosidase 2 subunit beta | PRKCSH | 1390246 | 3053446 | 0.4553 |
| A0A0V1BID7_TRISP | Rho GDP-dissociation inhibitor 1 | Arhgdia | 9136836 | 20262660 | 0.4509 |
| A0A0V1BT00_TRISP | Uncharacterized protein | T01_10834 | 23502019 | 52514259 | 0.4475 |
| E5S581_TRISP | Charged multivesicular body protein 5 | Tsp_06047 | 2987547 | 6734956 | 0.4436 |
| A0A0V1ATX6_TRISP | Charged multivesicular body protein 5 | T01_11566 | 2327660 | 5381135 | 0.4326 |
| E5SF13_TRISP | Zinc finger protein 364 | Tsp_02330 | 147262692 | 344313303 | 0.4277 |
| A0A0V1BJS3_TRISP | Uncharacterized protein | T01_10384 | 2705158 | 6458700 | 0.4188 |
| A0A0V1BIH7_TRISP | Uncharacterized protein | T01_7775 | 174024814 | 419717201 | 0.4146 |
| A0A0V1BER2_TRISP | UV excision repair protein RAD23-like protein B | Rad23b | 7044902 | 17331430 | 0.4065 |
| E5S720_TRISP | Putative WAP-type 'four-disulfide core | Tsp_10948 | 3058539 | 7543612 | 0.4054 |
| A0A0V1BIZ0_TRISP | Translocon-associated protein subunit gamma | SSR31 | 5965011 | 14813511 | 0.4027 |
| A0A0V1BV96_TRISP | GrpE-like protein 1, mitochondrial | GRPEL1 | 5768056 | 14611621 | 0.3948 |
| A0A0V1ARB7_TRISP | 5'-nucleotidase (EC 3.1.3.5) | F25B5.3 | 722313 | 1876381 | 0.3850 |
| A0A0V1AWQ2_TRISP | Secretory carrier-associated membrane protein | Scamp1 | 8420125 | 22183918 | 0.3796 |
| A0A0V1AZ55_TRISP | Phosphoenolpyruvate carboxykinase | PEPCK | 1493179 | 3981649 | 0.3750 |
| A0A0V1B2Q8_TRISP | Serine/threonine-protein kinase N2 | pkn2 | 2572659 | 6942075 | 0.3706 |
| A0A0V1BBX7_TRISP | Ribosomal protein L19 | RpL19 | 12738991 | 34474402 | 0.3695 |
| A0A0V1ASJ1_TRISP | Putative nuclear transport factor 2 | ran-4 | 26779730 | 72887439 | 0.3674 |
| A0A0V1AYH5_TRISP | Cathepsin B-like cysteine proteinase | CATB | 760065 | 2108459 | 0.3605 |
| A0A0V1BU34_TRISP | FAM203 family protein | T01_9696 | 333502 | 926168 | 0.3601 |
| E5SES8_TRISP | Putative ENTH domain protein | Tsp_02244 | 628456 | 1753409 | 0.3584 |
| A0A0V1BGE3_TRISP | Putative trans-2-enoyl-CoA reductase 1, mitochondrial | W09H1.5 | 7171985 | 20049842 | 0.3577 |
| E5SS72_TRISP | Gamma-glutamylcyclotransferase | chac2 | 1322856 | 3729050 | 0.3547 |
| A0A0V1C0L4_TRISP | Methionine--tRNA ligase, cytoplasmic | mrs-1 | 2591356 | 7436487 | 0.3485 |
| A0A0V1BFA9_TRISP | Lysocardiolipin acyltransferase 1 | Lclat1 | 3354480 | 9719210 | 0.3451 |
| A0A0V1BJX1_TRISP | Putative pterin-4-alpha-carbinolamine dehydratase | pcbd-1 | 2355822 | 6926937 | 0.3401 |
| E5SAI9_TRISP | Leucyl-tRNA synthetase, cytoplasmic | Tsp_00765 | 3719172 | 11145064 | 0.3337 |
| A0A0V1C2W4_TRISP | Hexosaminidase D | Hexdc | 490997 | 1494959 | 0.3284 |
| A0A0V1BA29_TRISP | Proteasomal ubiquitin receptor ADRM1 | ADRM1 | 24595358 | 75448347 | 0.3260 |
| A0A0V1BMT1_TRISP | Uncharacterized protein | T01_3439 | 195402218 | 601755739 | 0.3247 |
| A0A0V1C1K8_TRISP | Putative glutamine-tRNA ligase | ers-1 | 282153 | 869133 | 0.3246 |
| E5SK16_TRISP | Non-specific serine/threonine protein kinase | Tsp_09176 | 8605379 | 27299433 | 0.3152 |
| E5SMF1_TRISP | Putative JmjC domain-containing histone demethylation protein 2B | Tsp_10769 | 14730800 | 47017505 | 0.3133 |
| E5SLN4_TRISP | Uncharacterized protein | Tsp_08112 | 999635 | 3277505 | 0.3050 |
| A0A0V1B3C7_TRISP | NAD(P) transhydrogenase, mitochondrial | NNT | 362896 | 1307313 | 0.2776 |
| A0A0V1B0H9_TRISP | Protein arginine N-methyltransferase 6 | prmt6 | 891016 | 3287644 | 0.2710 |
| Q9B8A2_TRISP | Cytochrome c oxidase subunit 2 | COX2 | 17901928 | 69124783 | 0.2590 |
| E5S1B0_TRISP | Surfeit locus protein 4 | sft-4 | 5333634 | 21962620 | 0.2429 |
| A0A0V1BWL9_TRISP | High mobility group protein 1.2 | hmg-1.2 | 2797915 | 12034691 | 0.2325 |
| E5S9J0_TRISP | Putative U1 small nuclear ribonucleoprotein 70 kDa | SNRNP70 | 5005988 | 21923344 | 0.2283 |
| A0A0V1B4G3_TRISP | 26S proteasome non-ATPase regulatory subunit 8 | PSMD8 | 1197897 | 5822542 | 0.2057 |
| A0A0V1BZX6_TRISP | LanC-like protein 2 | LANCL2 | 322581 | 1975345 | 0.1633 |
| E5S8I9_TRISP | Uncharacterized protein | Tsp_00058 | 4368096 | 28399285 | 0.1538 |
| A0A0V1BBL6_TRISP | Fragile X mental retardation protein 1-like protein B | Fmr1 | 4703267 | 30979625 | 0.1518 |
