# Supplementary material for: Proteomic analysis of the response of Trichinella spiralis muscle larvae to exogenous nitric oxide
Source: PLoS One. 2018 Jun 5;13(6):e0198205. doi: 10.1371/journal.pone.0198205 (PMC5988324; doi:10.1371/journal.pone.0198205)
Supplement: S2 Table — (DOCX) [file pone.0198205.s002.docx]

Table S2. The primers used in this study

| Gene Name | Sequence (5′→3′) |
| --- | --- |
| FRMD5 | pU: 5′-AGAACCAACCGCTAATGGAA-3′ |
|  | pD: 5′-CTGCATTAAAACTGCGGTGA-3′ |
| CaMKII | pU: 5′-AGATCAGGGGCGTCTCTATG-3′ |
|  | pD: 5′-GTGACCGTATCCCATTCTGG-3′ |
| Gyg1 | pU: 5′-ACAGGGCAGTTTTGATGGAG-3′ |
|  | pD: 5′-TGTATGGCAAACGGTGAGAG-3′ |
| CUT-1 | pU: 5′-GAATACGACGCGATGAAGC-3′ |
|  | pD: 5′-GGTTCGGGAAAAACTGCATA-3′ |
| unc-9 | pU: 5′-TCCAACCTCTGTGGACTCTTG-3′ |
|  | pD: 5′-CCGGGATCCAGGTACAGTTA-3′ |
| Fmr1 | pU: 5′-CGAAGTGAAAGGGCTTACCAC-3′ |
|  | pD: 5′-ACGCTCACGAGCTTGATACA-3′ |
| COX2 | pU: 5′-AGGAACCTTGTTCACCTCCA-3′ |
|  | pD: 5′-GTACGCCGAGTGGTGAGATT-3′ |
| PSMD8 | pU: 5′-TGATTTGGAAGTGGCACAAA-3′ |
|  | pD: 5′-CGCTATTGCTTCCCTAGCAC-3′ |
| Sft-4 | pU: 5′-AGATACACAGTCGGCATCGAG-3′ |
|  | pD: 5′-GCTTCACCGCTTTCAAATTC-3′ |
| hmg-1.2 | pU: 5′-AAGAAATGTTCCGCGAAATG-3′ |
|  | pD: 5′-TTCCAATTCAGCATCGTAGC-3′ |
| GAPDH | pU: 5′-TGGCTTAGCTCCGTTGG-3′ |
|  | pD: 5′-TTTGGGTTGCCGTTGTA-3′ |
