# Supplementary figures and images for: Proteomic analysis of the response of Trichinella spiralis muscle larvae to exogenous nitric oxide
Source: PLoS One. 2018 Jun 5;13(6):e0198205. doi: 10.1371/journal.pone.0198205 (PMC5988324; doi:10.1371/journal.pone.0198205)

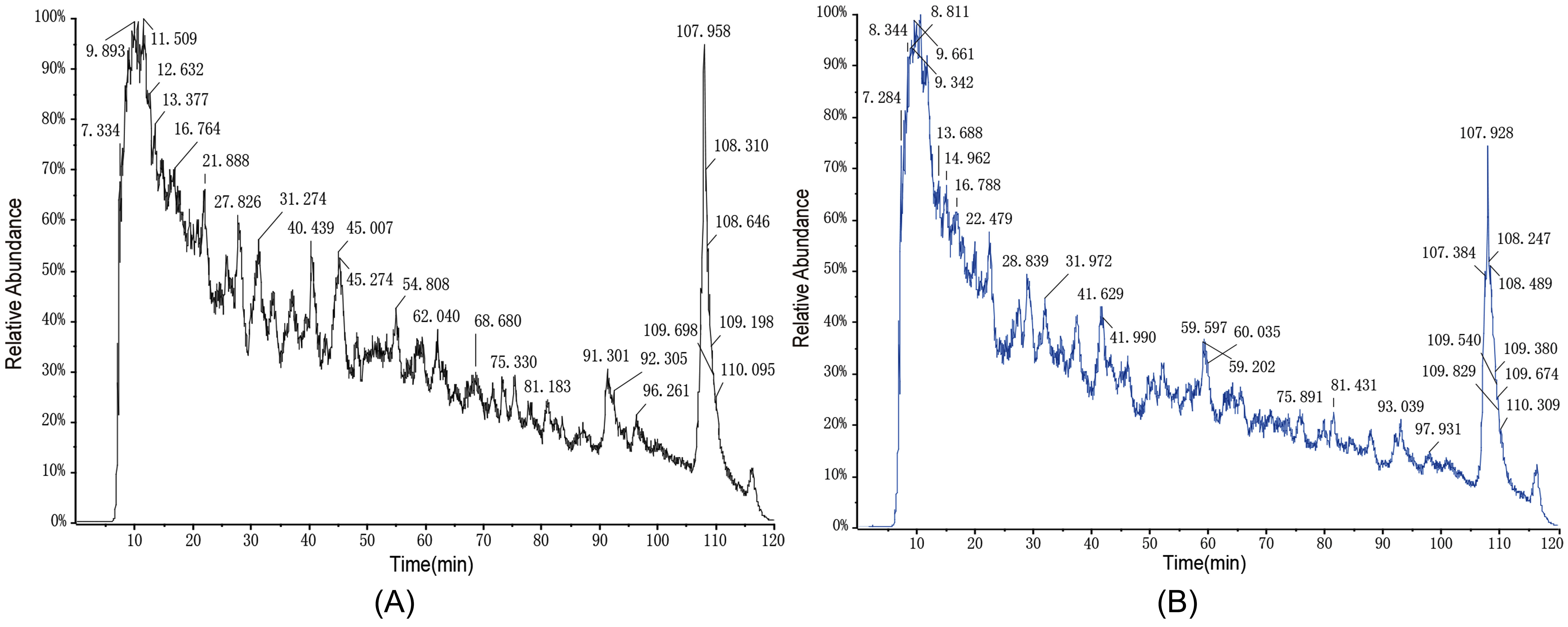

Supplement: S1 Fig — (A) Proteins expressed in the control group; (B) Proteins expressed in the sodium nitroprusside treatment group. The images indicated that several differential peaks were identified in T. spiralis muscle larvae in response to NO stress. (TIF) [file pone.0198205.s003.tif]

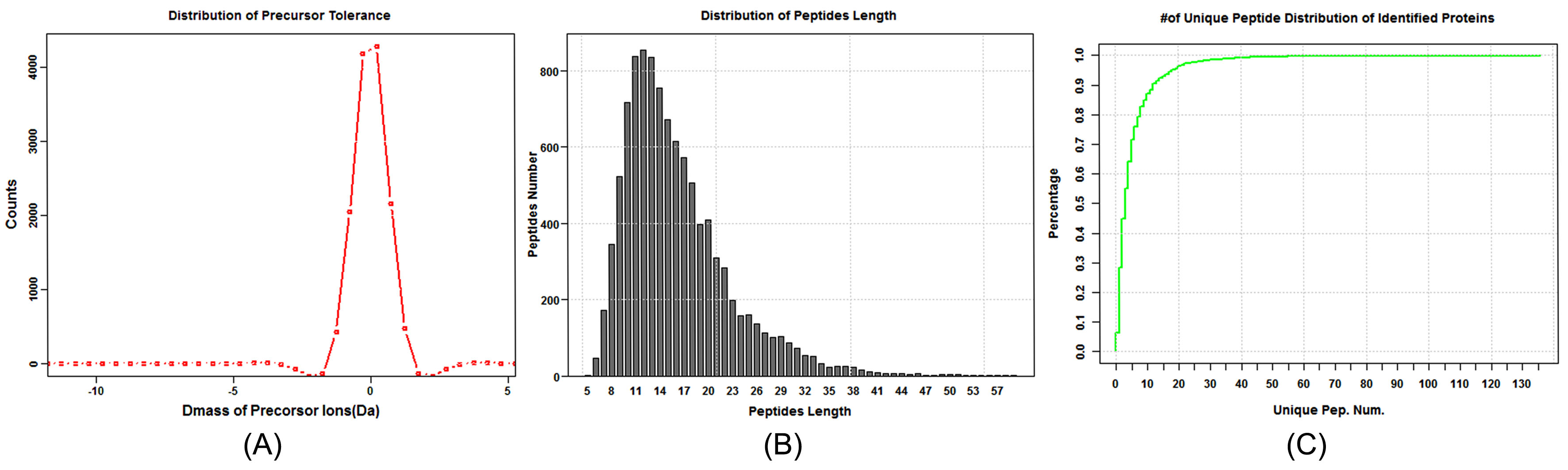

Supplement: S2 Fig — (A) Distribution of precursor tolerance. (B) Distribution of peptides length. (C) Unique peptide distribution of the identified proteins. The images indicated that the quality tolerance was in the range of ±0.1 Da, and peptides length was focused on the range of 8–22. (TIF) [file pone.0198205.s004.tif]

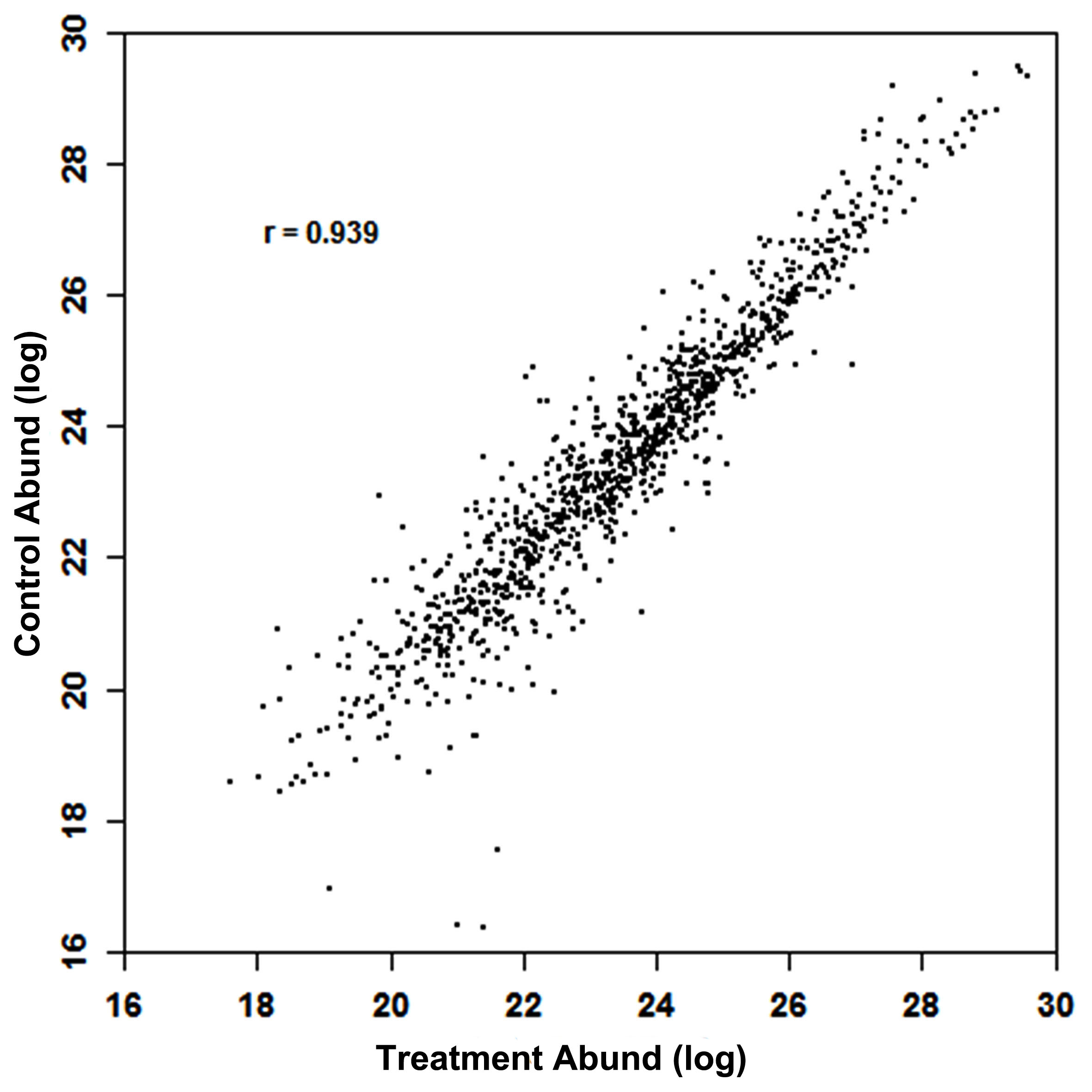

Supplement: S3 Fig — The images indicated that the r of Pearson's correlation was 0.939, and had a higher correlation between the control and treatment groups. (TIF) [file pone.0198205.s005.tif]

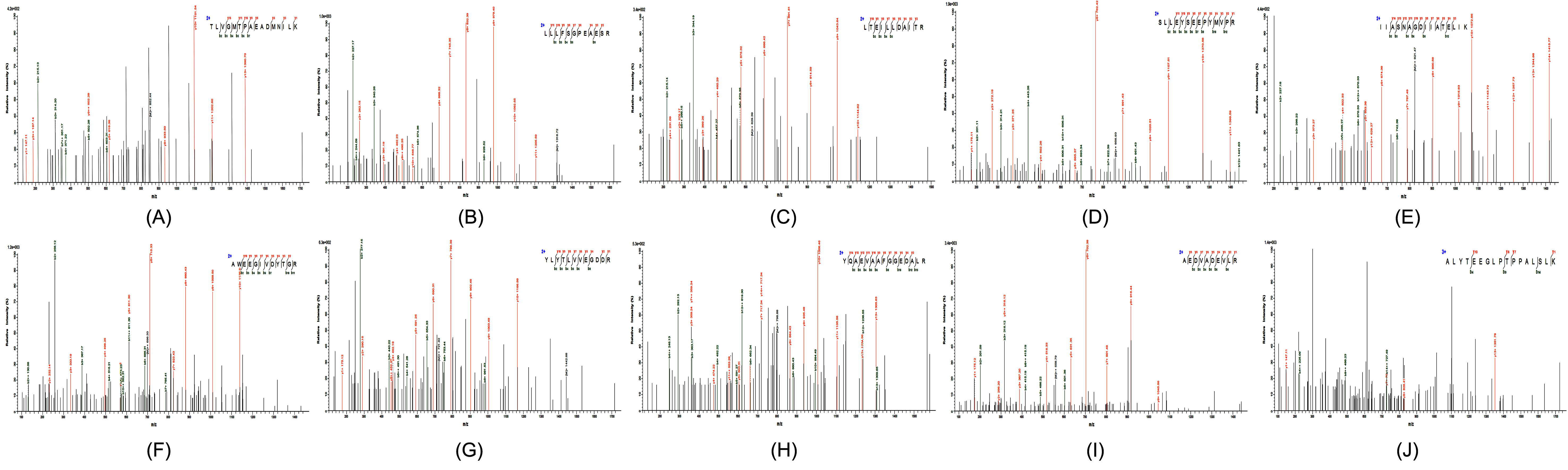

Supplement: S4 Fig — Secondary mass spectrogram of five expressed proteins in the control (A–E) and the treatment groups (F–J), respectively. (TIF) [file pone.0198205.s006.tif]
